# Supplementary material for: Variations in brain defects result from cellular mosaicism in the activation of heat shock signalling
Source: Nat Commun. 2017 May 2;8:15157. doi: 10.1038/ncomms15157 (PMC5418582; doi:10.1038/ncomms15157)
Supplement: Supplementary Information — Supplementary Figures, Supplementary Tables and Supplementary References. [file ncomms15157-s1.pdf]

Supplementary Figure 1

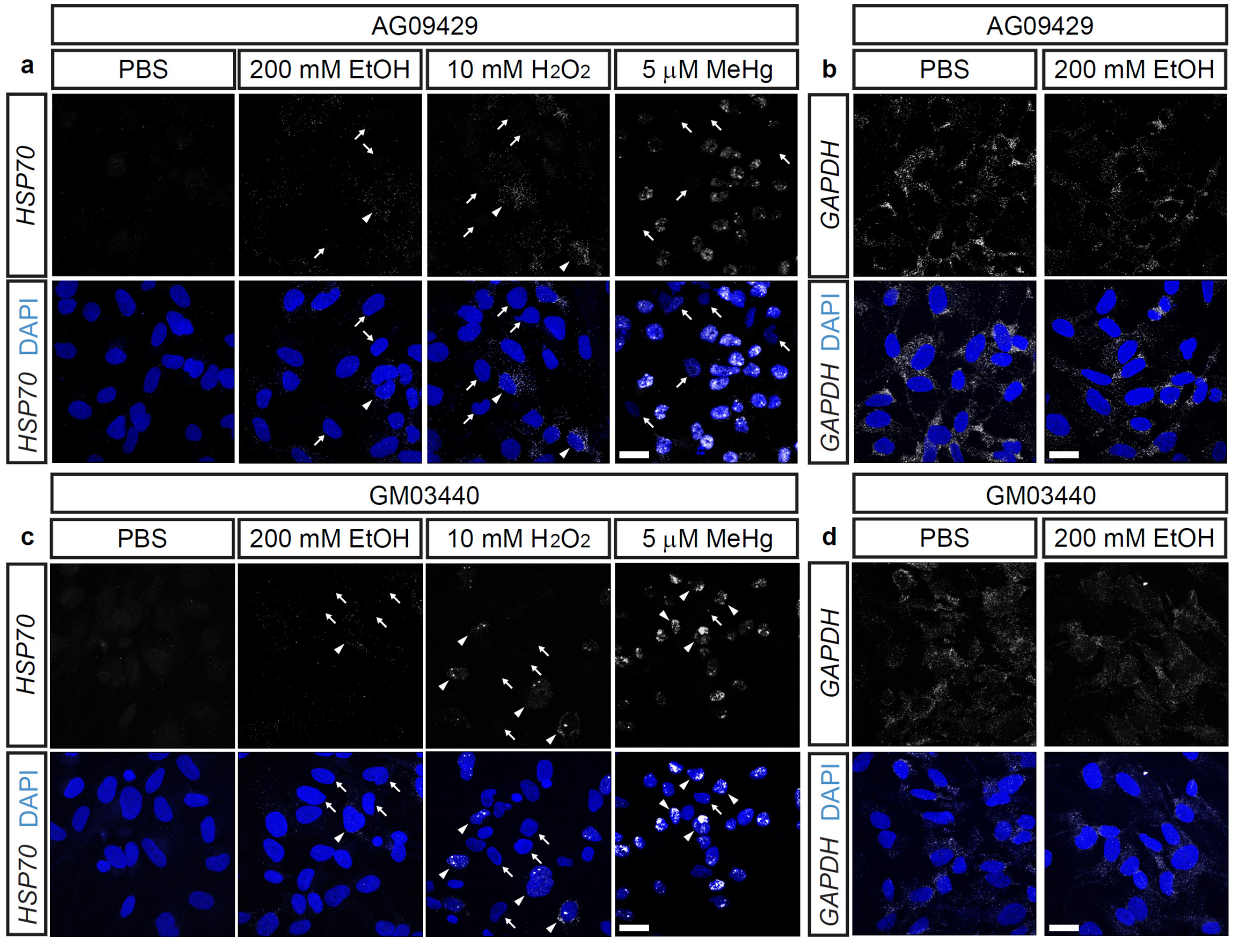

Supplementary Figure 1| Variable levels of activation of HSF1-HSP signaling by environmental stress.

(a-d) Representative images of smFISH. *HSP70* (a,c) shows heterogeneous expression in response to exposure to the indicated reagents in two hNPC lines (AG09429 and GM03440). Several cells have abundant *HSP70* mRNA particles as shown in white arrowheads, while neighboring cells have few particles as shown in white arrows. *GAPDH* (b,d) shows homogeneous expression in exposure to 200 mM EtOH in two hNPC lines. Bars = 0.02 mm.

Supplementary Figure 2

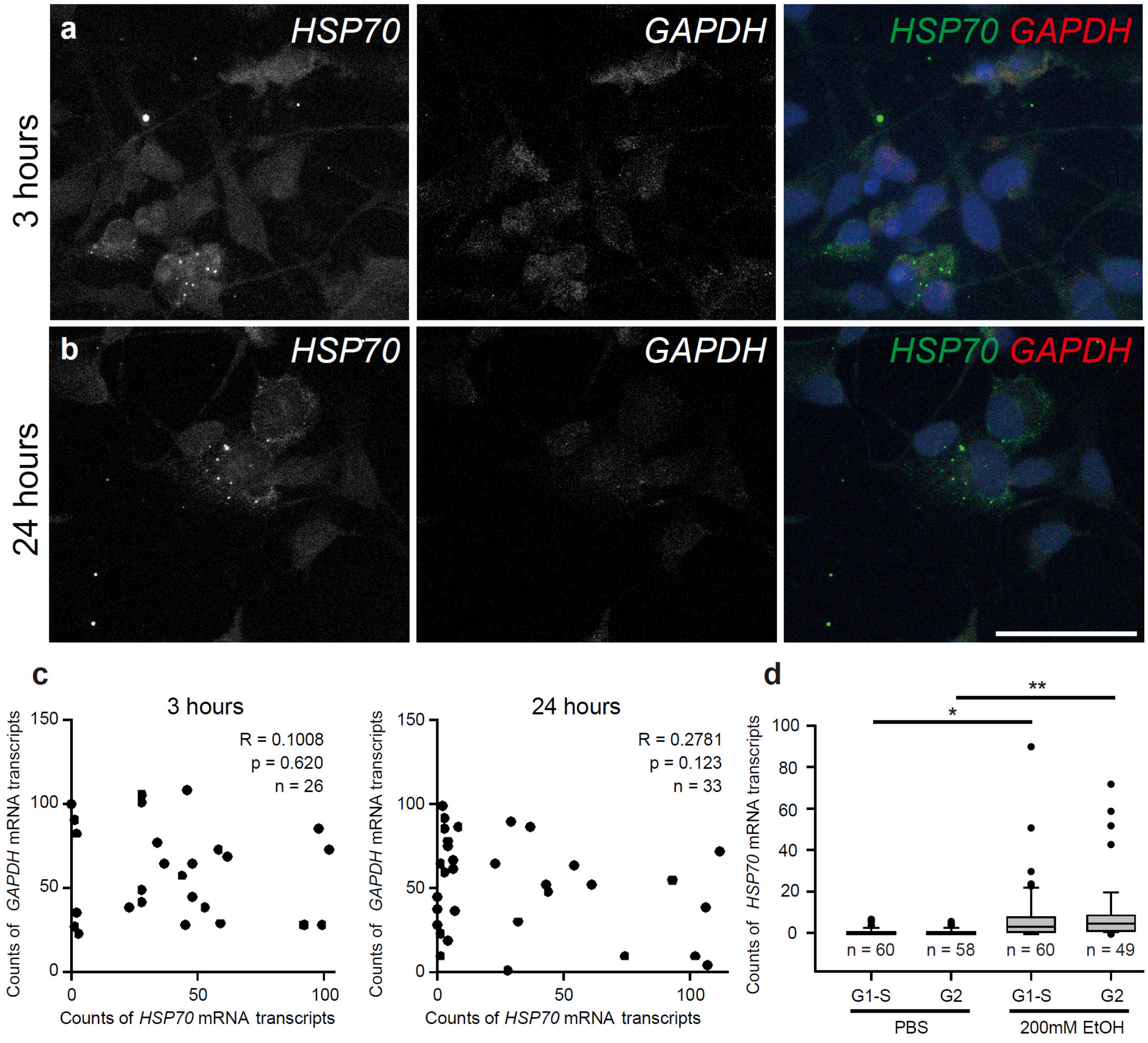

Supplementary Figure 2| No correlation between the expression levels of *HSP70* and *GAPDH*. (a,b) Representative images of *HSP70* and *GAPDH* double smFISH at the indicated times of exposure to 200 mM EtOH. Bar = 0.05 mm. (c) The numbers of *HSP70* and *GAPDH* mRNA particles in each cell show no correlation at either 3 hours and 24 hours exposure of EtOH (Pearson's correlation analysis), suggesting that the mosaicism of *HSP70* expressions is not due to cellular changes in global transcriptions. Note that variability in *HSP70* mRNA expressions was still sustained after the 24 hours exposure. The overall expression levels of both

GAPDH and *HSP70* were slightly decreased. (d) Quantification of *HSP70* mRNA puncta in G1-S and G2 phases (defined by co-labeling pattern of Ki67 immunocytochemistry) in hNPCs exposed to PBS or 200 mM EtOH for 3 hours. No difference was seen in the variability of *HSP70* mRNA levels between G1-S and G2 ($p>0.05$ by Levene's test), while the variability in *HSP70* mRNA levels was seen at G1-S and G2 phase between PBS and EtOH exposure (*$p<0.005$, **$p<0.001$ by Levene's test). More than 40 cells in different positions from multiple dishes were measured per group.

Supplementary Figure 3

a

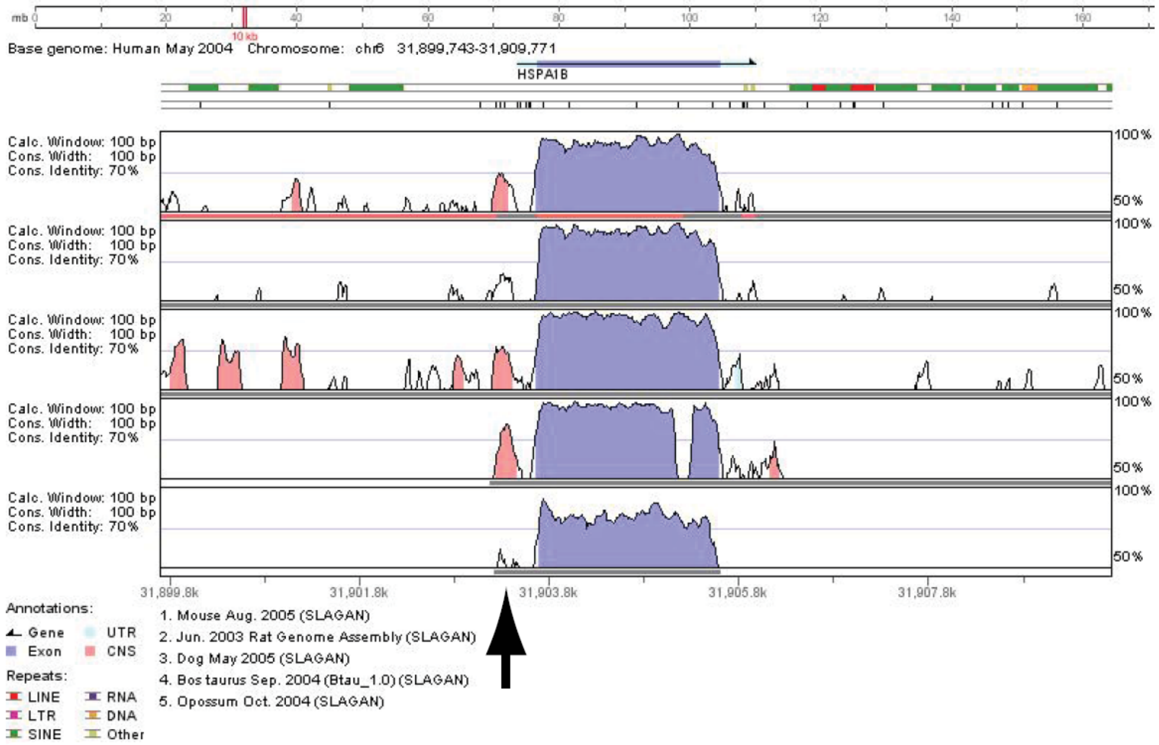

b

```
>mouse HSE full sequence (649bp)
CAGCTTCACCCACAGGGACCCCGAAGTTGCGTCGCCTCCGCAACAGTGTC AATAGCAGCACCAGCACTTCCCCACACCCTCCCCCTCAGGAATCCGTACT
CTCTAGCGAACCCCAGAAACCTCTGGAGAGTTCTGGACAAGGGCGGAACC CACAACTCCGATTACTCAAGGGAGGCGGGGAAGCTCCACCAGACGCGAAA
CTGCTGGAAGATTCCTGGCCCCAAGGCCTCCTCCGGCTCGCTGATTGGCC CAGCGGAGAGTGGGCGGGGCCGGTGAAGACTCCTTAAAGGCGCAGGGCGG
CGAGCAGGGCACCAGACGCTGACAGCTACTCAGAATCAAATCTGGTTCCA TCCAGAGACAAGCGAAGACAAGAGAAGCAGAGCGAGCGGCGCGTTCCCGA
TCCTCGGCCAGGACCAGCCTTCCCCAGAGCATCCACGCCGCGGAGCGCAA CCTTCCCAGGAGCATCCCTGCCGCGGAGCGCAACTTTCCCCGGAGCATCC
ACGCCGCGGAGCGCAGCCTTCCAGAAGCAGAGCGCGGCGCCATGGCCAAG AACACGGCGATCGGCATCGACCTGGGCACCACCTACTCGTGCGTGGGCGT
GTTCCAGCACGGCAAGGTGGAGATCATCGCCAACGACCAGGGCAACCGC

>mouse mutHSE full sequence (649bp)
CAGCTTCACCCACAGGGACCCCGAAGTTGCGTCGCCTCCGCAACAGTGTC AATAGCAGCACCAGCACTTCCCCACACCCTCCCCCTCAGGAATCCTTACT
CTTTATCGAACCTCATAAACCTTTGTAGAGTTTTGGACAAGGGCGGAACC CACAACTCCGATTACTCAAGGGAGGCGGGGAAGCTCCACCAGATGCTAAA
CTGTTGTAAGATTTCTTGCCCCAAGGCCTCCTCCGGCTCGCTGATTGGCC CAGCGGAGAGTGGGCGGGGCCGGTGAAGACTCCTTAAAGGCGCAGGGCGG
CGAGCAGGGCACCAGACGCTGACAGCTACTCAGAATCAAATCTGGTTCCA TCCAGAGACAAGCGAAGACAAGAGAAGCAGAGCGAGCGGCGCGTTCCCGA
TCCTCGGCCAGGACCAGCCTTCCCCAGAGCATCCACGCCGCGGAGCGCAA CCTTCCCAGGAGCATCCCTGCCGCGGAGCGCAACTTTCCCCGGAGCATCC
ACGCCGCGGAGCGCAGCCTTCCAGAAGCAGAGCGCGGCGCCATGGCCAAG AACACGGCGATCGGCATCGACCTGGGCACCACCTACTCGTGCGTGGGCGT
GTTCCAGCACGGCAAGGTGGAGATCATCGCCAACGACCAGGGCAACCGC
```

Supplementary Figure 3| Sequences of HSE and mutHSE.

(a) Comparative analysis of the human *Hsp70* genomic region with that of indicated species using the Vista Genome Browser. The arrow indicates the presumptive promoter of *Hsp70* that is conserved across species. (b) Full sequences of HSE and mutHSE. Pink letters indicate HSF binding domains and blue letters indicate point mutations in them. The details of reporter evaluation are found in [1].

Supplementary Figure 4

a

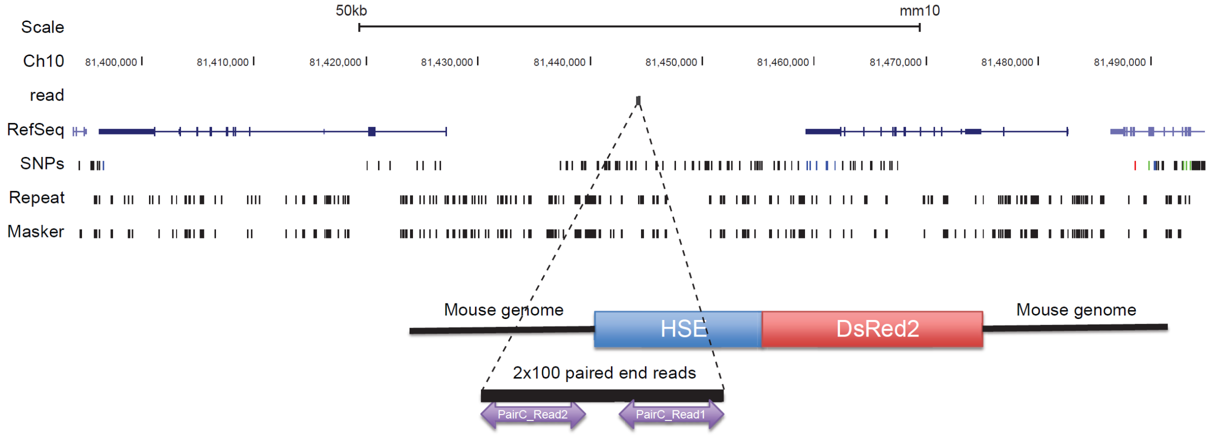

PairC_Read1HWI-ST1318:395:H2VHGBCXY:2:2209:7756:99552
CTTCCCCACACCCTCCCCCTCAGGAATCCGTACTCTCTAGCGAACCCCAGAAACCTCTGGAGAGTTCTGGACAAGGGCGGAACC
CACAACTCCGATTACTC
HSE

PairC_Read2
CTACAGACACCTAGAGGAACCTACAGACACTTATAGACTCCTACAGACACCTACAGATTCCTACTGATTTTTACACACACCCATAGA
TGGCTACAGAAATC
chr10 intergenic

b

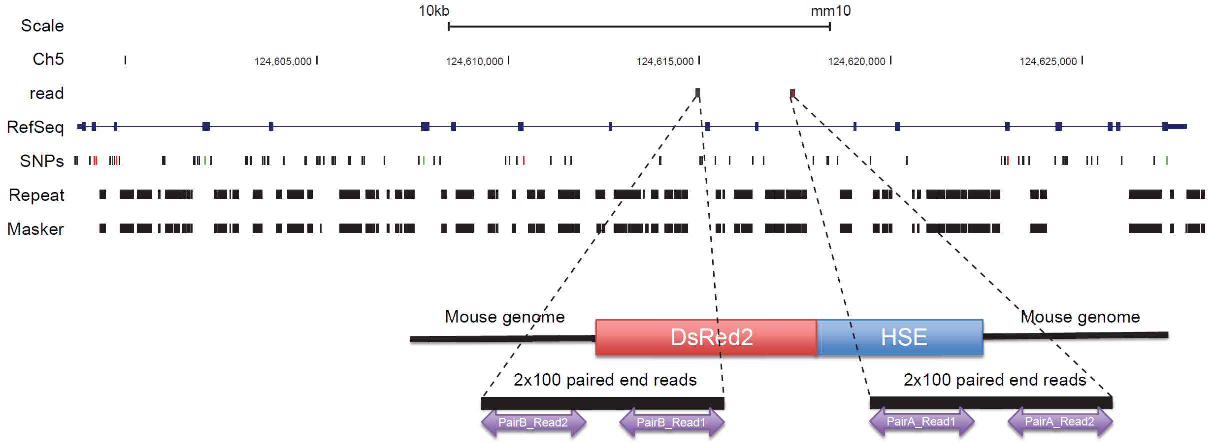

PairB_Read1 HWI-ST1318:395:H2VHGBCXY:2:1201:4509:3972
CATCGTGGAGCAGTACGAGCGCACCGAGGGCCGCCACCACCTGTTCCTGTAGCGGCCGCGACTCTAGATCATAATCAGCCATACCAC
TGTAGAGGTTT DsRed2
PairB_Read2 HWI-ST1318:395:H2VHGBCXY:2:1201:4509:3972
GTGCTCAAGCTTTGAGCCAAGCACTGGGGACGCGGGAATCAAAGGAAAGGGACAGAGTTATTCAGGGAACTGAGGTGGAATAGAGGA
GGCCGACCCTG chr5 intron of Tctn2 gene

PairA_Read1 HWI-ST1318:395:H2VHGBCXY:2:2105:15907:13439
CCCAAGGCCTCCTCCGGCTCGCTGATTGGCCCAGCGGAGAGTGGGCGGGGCCGGTGAAGACTCCTTAAAGGCGCAGGGCGGCGAG[illegible]
GGCACCAGACGCT HSE
PairA_Read2 HWI-ST1318:395:H2VHGBCXY:2:2105:15907:13439
CAGGGCCTCTGGAAGAGCAGCCACTGCTCTTAACTGCTGAGGCATCTCTCCAGCCCCTTGTTTAAACATTATATTTATTTATTTATTTGT
TGTGTGTT chr5 intron of Tctn2 gene

Supplementary Figure 4| Genomic loci of transgene integration in HSE-RFP Tg mice.

(a) NGS whole exome sequencing with 3X coverage defined the intergenic domain in

chromosome 10 as the region of the transgene insertion. The 200-base-pair read includes a partial sequence of HSE. (b) Another integrated region was found in the *Tctn2* gene locus on chromosome 5. The transcriptional direction of the transgene is opposite to that of *Tctn2* gene.

Supplementary Figure 5

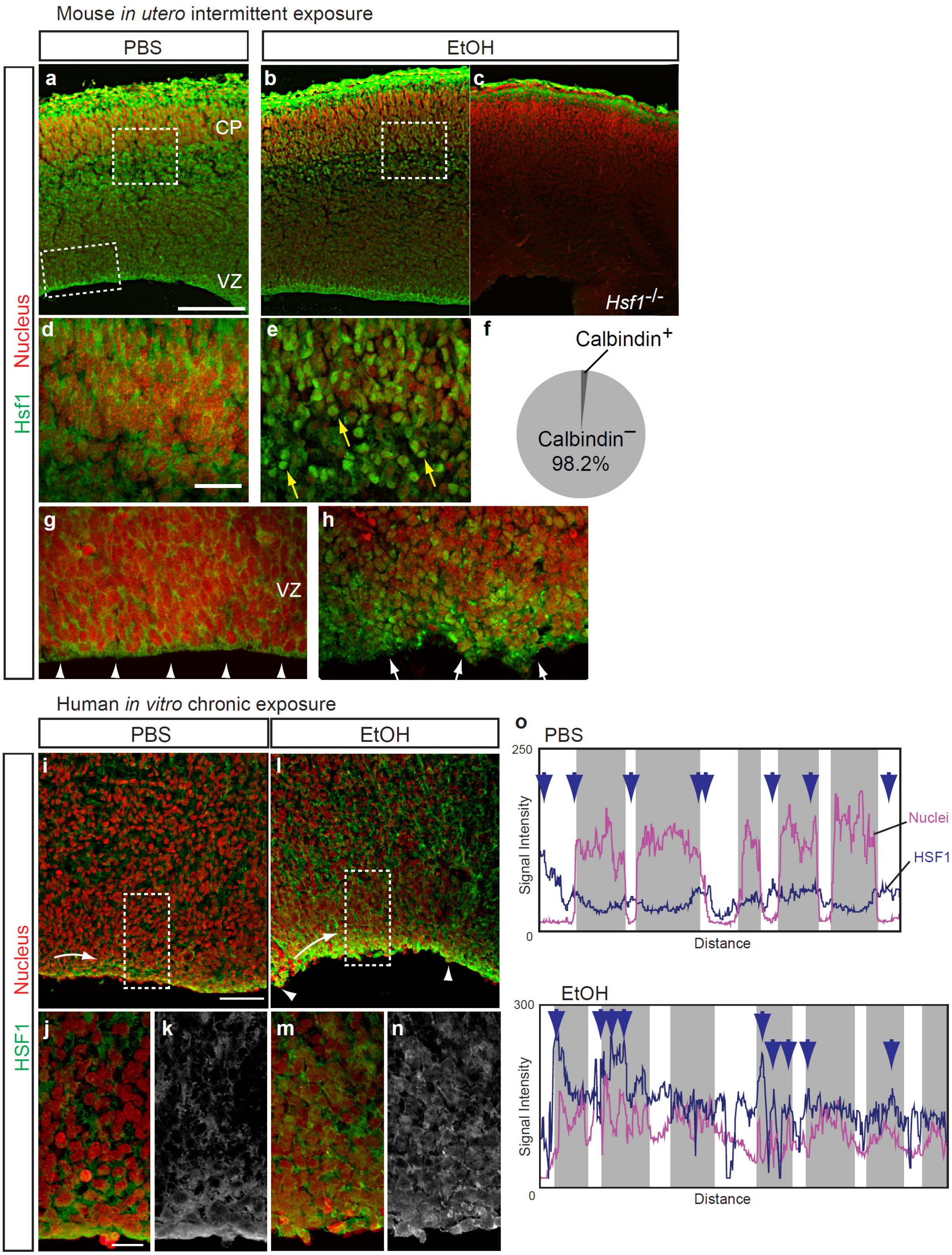

Supplementary Figure 5| Nuclear translocation of HSF1 in prenatal mouse and human cortices exposed to EtOH.

(a-h) Cortical slices from PBS- (control, a) or EtOH-exposed wild-type (b) and *Hsf1* KO (c) mouse embryos were immunostained for Hsf1 (green) with nuclear counter-stain (red) at E16. d, e, g are higher magnification views of the boxed regions in a and b. h is from another sample of EtOH-exposed wild-type embryo. In the cortex exposed to EtOH, strong nuclear labeling of Hsf1 is observed in subpopulations of cortical cells, e.g., immature neurons below the cortical plate (CP) (arrows in e) and NPCs in the ventricular zone (VZ) where the ventricular surface is disrupted (arrows in h). The white arrowheads in g indicate apically polarized expression of Hsf1 in the intact VZ in the control (n=8/8 embryos). (f) The ratio of calbindin^{+} inhibitory and calbindin^{-} excitatory neurons in the neurons with nuclear localization of Hsf1 in the cortical plate at E16. Activation of Hsf1-Hsp signaling occurred mostly in excitatory neurons in the cortex. Nuclear translocation of Hsf1 in the intermediate zone was observed even 48 hours after EtOH injection (n=3/3), consistent with the prolonged activation of Hsf1 in the EtOH-exposed embryonic cortex[2]. Scale Bar = 250 µm. (i-n) Gestational week (GW) 15 human cortical slices cultured with PBS (i-k) or EtOH (at 50 mM, 24 hours) containing media (l-n) were immunostained for HSF1 (green) with nuclear counter-stain (red). j, k and m, n are higher magnification views of the squared regions in i and l, respectively. Consistent with the rodent model, the nuclear distribution of HSF1 was more evident around moderately dysaligned ventricular surface (e.g. region between arrowhead) likely caused by EtOH exposure[3] (m,n), whereas apically polarized HSF1 expression was observed in the VZ in the control (j,k). Bar = 100 µm (i, l), 25 µm (j, k, m, n). (o) Quantification of the fluorescent signal intensity (signal intensities of HSF1 and nuclear labeling were measured along the white arrows in the VZ in i

and l) shows higher expression of HSF1 in the nuclei relative to the cytoplasm, as well as an overall increase of HSF1 expression, in the EtOH-exposed cortex (n=4/4 brains). Arrows indicate the peaks of HSF1 expression (blue lines). In the EtOH-exposed cortex, the peaks are accumulated within the domains where nuclear staining (pink lines) shows higher intensity (highlighted in gray). n= 6 slices from one GW 15 and one GW 18 cortices (3 slices each).

Supplementary Figure 6

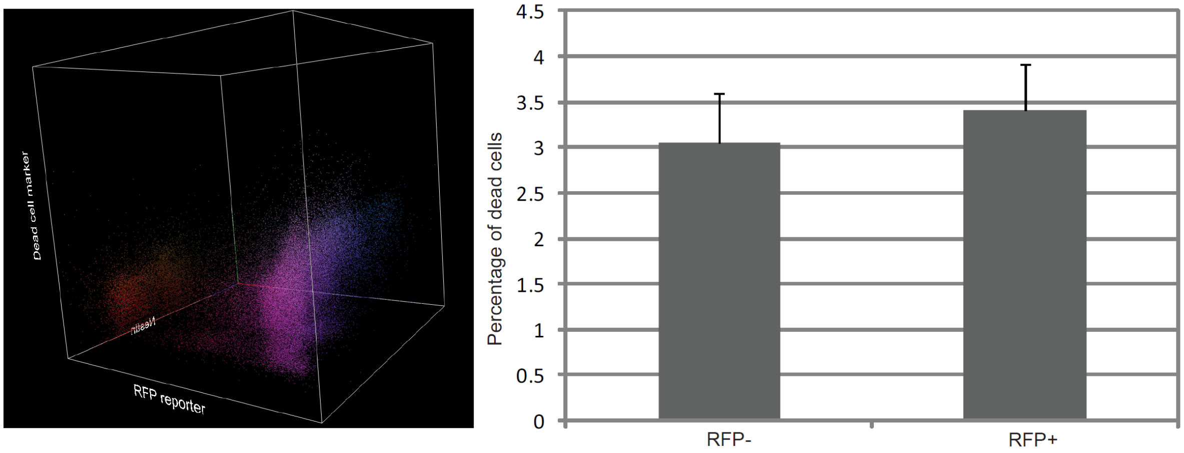

Supplementary Figure 6| Reporter-expressing cells in the cortex of HSE-RFP reporter transgenic mice are living cells.

Representative FACS analysis shows no significant differences in the percentage of cells expressing dead cell marker between reporter-positive and -negative cells. After dissociation from the reporter-expressing cortical region at E16 post-exposure to daily intermittent EtOH regimen, cells were stained with Nestin and dead cell marker. Left: Nestin$^-$/RFP$^-$ (orange), Nestin$^+$/RFP$^-$ (red), Nestin$^-$/RFP$^+$ (blue) and Nestin$^+$/RFP$^+$ (purple) cells are plotted according to the expression of RFP (X-axis), Nestin (Y-axis) and dead cell marker (Z-axis). Right: The percentage of cells expressing dead cell marker was quantified. Data are represented as mean ± SEM. *p=n.s.* by t-test (n=3 each).

Supplemetary Figure 7

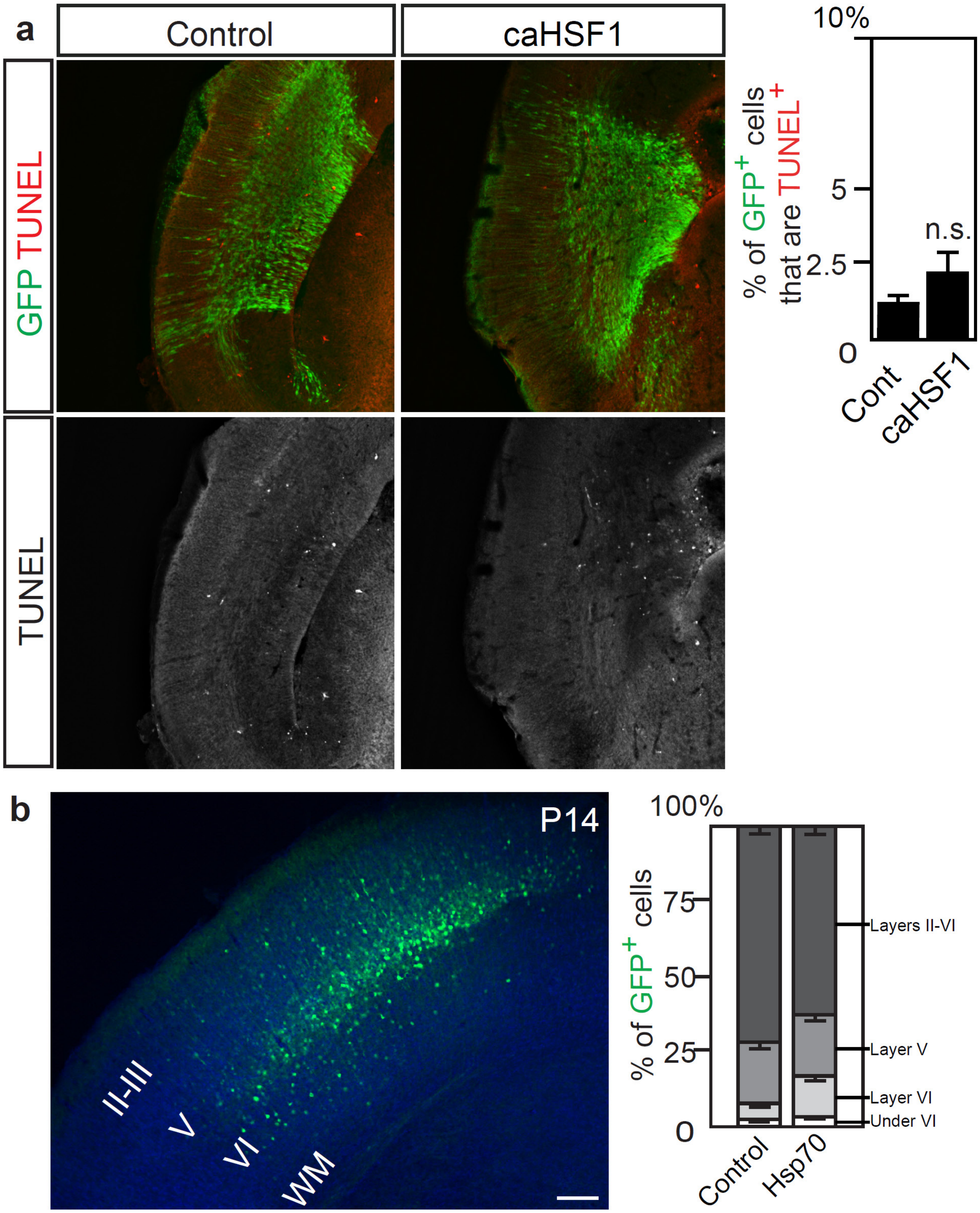

Supplementary Figure 7| caHSF1 or Hsp70 overexpression does not induce apoptosis or migration defects, respectively.

(a) Representative images of TUNEL staining (red, white) at E16 on control- and caHSF1-electroporated (at E14) cortices, and the quantification of $TUNEL^+$ cells in the electroporated (GFP^+) cells. Data are represented as mean ± SEM. *p=n.s* by t-test. (b) Unlike caHSF1 electroporation (Fig.5), *Hsp70* overexpression (IUE at E14) did not produce heterotopia and showed little effects on the radial migration of the cortical neurons as observed at P14 (*p=n.s.*, repeated measures ANOVA). Data are represented as mean ± SEM. It contrasts with the role of Hsp70 in mediating the function of Hsf1 in neuroprotection[4]. Bars = 0.2 mm.

Supplementary Figure 8

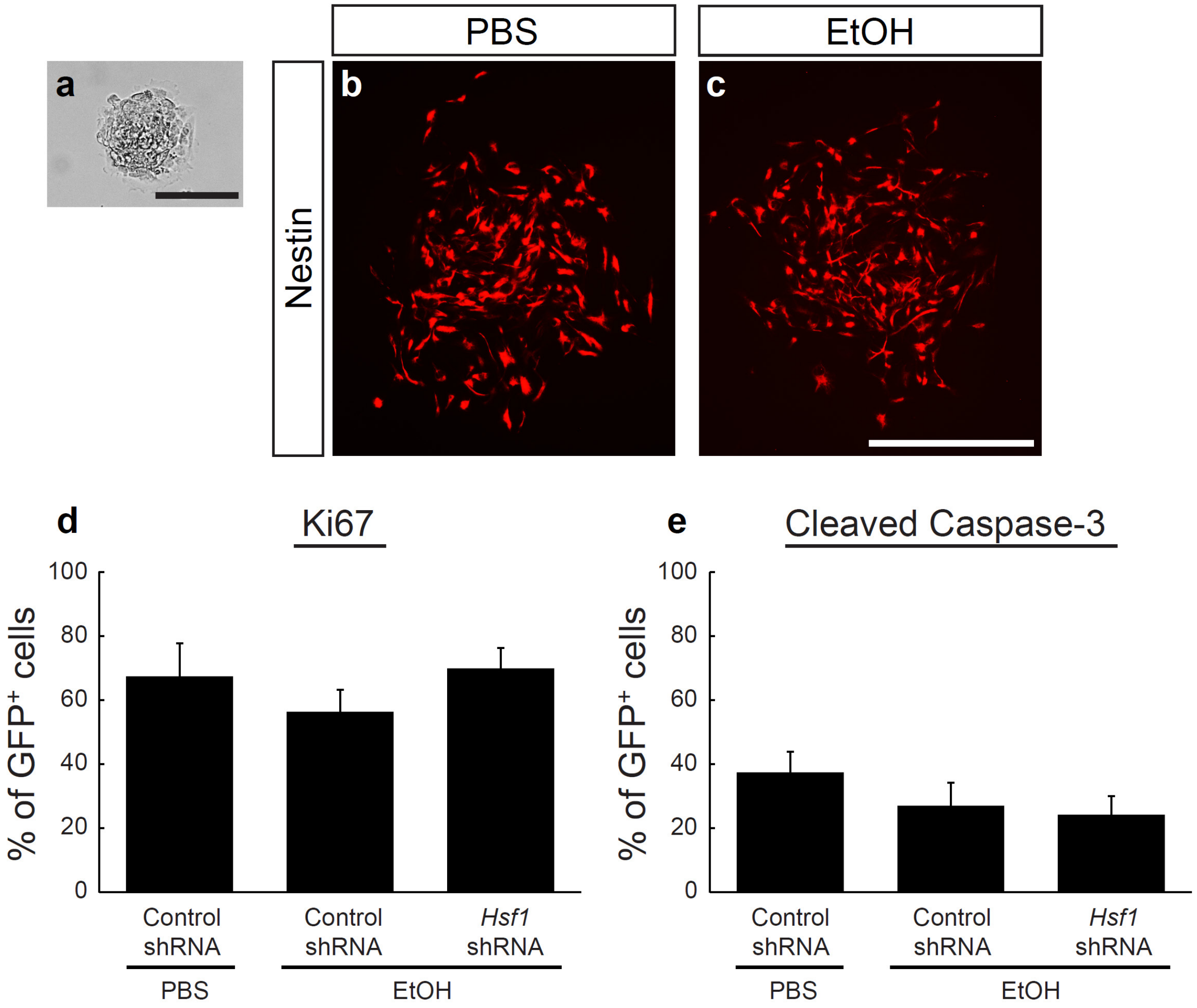

Supplementary Figure 8| EtOH exposure or knockdown of *Hsf1* does not affect proliferation and apoptosis of NPCs *in vitro*.

(a) A representative image of a neurosphere cultured for 30 minutes on an adhesive bottom for the migration assay. The cells extend processes but have not migrated out of the neurosphere yet. Scale Bar = 0.1 mm. (b,c) Similar numbers of Nestin$^+$ cells were observed in the neurospheres exposed to PBS (b) or EtOH (c) for 3 hours for the migration assay. Bars = 0.2 mm. (d,e) The percentages of Ki67$^+$ (d) and active Caspase-3$^+$ (e) cells in the total number of GFP$^+$ cells. Data are represented as mean ± SEM. One-way ANOVA, $F(2,9)=0.81$, $p=0.475$, and $F(2,9)=1.16$, $p=0.356$, respectively. Note that, regardless of migration distance, the percentage of the cells

expressing those markers does not differ, suggesting that effects on the migration is unlikely secondary effects of cell proliferation/differentiation and death (One-way ANOVA, $F(2,9)=0.67$, $p=0.535$ for Ki67, $F(2,9)=0.97$, $p=0.415$ for active Caspase-3).

Supplementary Figure 9

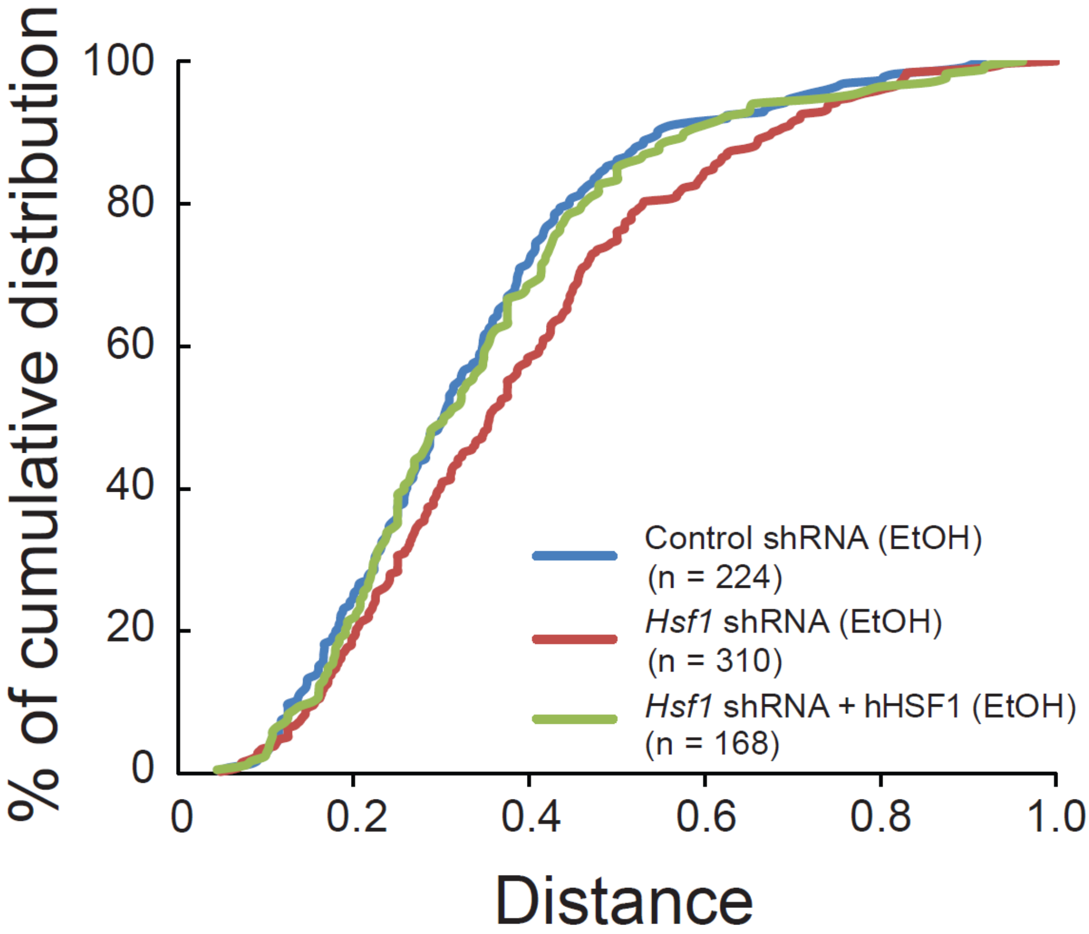

Supplementary Figure 9| Overexpression of human HSF1 abolishes *Hsf1* shRNA-mediated amelioration of EtOH-induced migration defects.

Cell migration assay of NPCs, showing the cumulative distribution of cells after a 6-hour culture (see Fig.8). The data for Control shRNA (EtOH)(blue) and *Hsf1* shRNA (EtOH)(red) are the same as those shown in Fig.8f. The alleviation of EtOH-induced migration deficits by *Hsf1* shRNA is abolished by co-introduction of full-length human HSF1 (Hsf1 shRNA + hHSF (EtOH)(green). $p<0.05$ by K-S test for each of comparisons except that between Control shRNA (EtOH) vs *Hsf1* shRNA (EtOH).

Region	Embryo	% of RFP+ in GFP+ electroporated cells	Average	Difference in distribution (migration) of RFP+ and RFP- cells (p value by K-S test)
Dorsofrontal	1	32	24.40+/-5.848	0.09
	2	12.9		0.47
	3	28.3		0.33
Mediofrontal	1	27.2	25.43+/-2.688	0.09
	2	19.6		0.58
	3	22.9		0.02
	4	32		0.73
Lateral	1	18.2	23.58+/-3.131	0.93
	2	12.1		0.08
	3	29.8		0.27
	4	32.9		0.24
	5	26.1		0.79
	6	22.4		0.21
Medial	1	18	19.25+/-2.315	0.22
	2	15.2		0.68
	3	28.5		0.08
	4	23		0.27
	5	18		0.24
	6	12.8		0.79
Mediocaudal	1	32.5	27.88+/-4.928	0.23
	2	22.6		0.97
	3	39.2		0.1
	4	17.2		0.79

Supplementary Table 1| Reporter analysis of different cortical regions.

The table shows the percentages of reporter expressing cells in total electroporated cells in indicated cortical areas in each animal after daily administration of EtOH until E16. There are no differences in the average percentages of reporter-positive cells across cortical areas (*p=0.5483*, Kruskal-Wallis test). For all animals, comparison of radial distribution patterns of the reporter-positive and -negative cells was made by quantifying the percentage of the cells in each of 10 bins defined along radial axis, and the K-S test on the distribution pattern showed no significant differences between reporter-positive and -negative cells**.**

Mouse HSP70 probes		Human HSP70 probes
Sequences 1	Sequences 2	Sequences 1
atgccgatcgccgtgttctt	tggtcgttggcgatgatctc	accttgccgtgttggaacac
accttgccgtgctggaacac	ttcgcgtcgaacacggtgtt	acctggttcttggccgcatc
ctggtcgttggcgatgatct	agatctcctccgggaagaac	gtcaaacacggtgttctgcg
tcggtgaaggccacgtagct	atctccttcatcttcgtcag	ccgaacttgcggccaatcag
cgtctccgatgaggcgctcg	cgctgagagtcgttgaagta	cagtgcttcatgtccgactg
gtcgaacacggtgttctgcg	atgatccgcagcacgtttag	gtcgttgatcacctggaaag
ccgaacttgcggccgatcag	aggtcgaagatgagcacgtt	ttgtagctcacctgcacctt
ggcttgtcgccgtcgttcac	tcaggatggacacgtcgaac	accatggacgagatctcctc
cttgtagttcacctgcacct	ctgagcacagctcttcgaac	ggtagcccaggtacgcctcg
ggaagaacgaccggctctcg	ttgaagaagtcctgcagcag	gtgatcaccgcgttggtcac
accatggacgagatctcctc	ttgatgctcttgttcaggtc	cgctgcgagtcgttgaagta
agcgatctccttcatcttcg	gagtaggtggtgaaggtctg	ctcgttgatgatccgcagca
gtgatcaccgcgttggtcac	tcgtacacctggatcagcac	cgttctgtccaggccgtagg
cgctgagagtcgttgaagta	atgtcgaaggtcacctcgat	ggtcaaagatgagcacgttg
cacgtttagaccggcgatca	tgacgttcaggatgccgttg	tcgatcgtcaggatggacac
cgtgggctcgttgatgatcc	cagcaccttcttcttgtcag	tggccttcacctcgaagatg
gtcgaagatgagcacgttgc	cagtgcttcatgtcggactg	agcctgttgtcaaagtcctc
tcgatcgtcaggatggacac	ttgttctggctgatgtcctt	ctcacggctcgcttgttctg
tccacgaagtggctcaccag	ccctcgaacagagagtcgat	ctggacgacagggtcctctt
gcaccgcgcgcttgttctgg	cgcgtgatggatgtgtagaa	tcggagcacagctcctcgaa
ctggacgacagcgtcctctt	gtggagttgcgcttgatgag	ctccagggtgcttcggaaca
ctgagcacagctcttcgaac	ttggtgatggtgatcttgtt	ccttgtccagcttggcgtcg
tccagcgtgccgcggaacag	cataggattcgagcgcgttc	aggaccaggtcgtgaatctg
ccttgtccatcttggcgtcg	acggcgctcttcatgttgaa	agcttctgcaccttggggat
agcaccaggtcgtggatctg	tggtacagcccactgatgat	cccgttgaagaagtcctgca
agcttctgcaccttggggat	ttgaactcctccacgaagtg	ttgatgctcttgttcaggtc
cccgttgaagaagtcctgca		tgcacgttctcggacttgtc
ttgatgctcttgttcaggtc		cttgatcagggcagtcatca
catcaggatggccgcctgca		tgaagatctgcgtctgcttg
tgcacgttctccgacttgtc		ctcgtacacctggatcagca
cttgatgagcgccgtcatca		ttgttgtctttcgtcatggc
ctcgtacacctggatcagca		ggatgccgctcagctcgaag
aggttgttgtcgcgcgtcat		catcgatgtcgaaggtcacc
ggatgccgctcagttcgaag		gtgacgttcaggatgccgtt
gaaggtcacctcgatctgtg		atggtgatcttgttggcctt
tgacgttcaggatgccgttg		ggcgctcttcatgttgaagg
cttgccggtgctcttgtcgg		tcgctgatcttgcccttgag
ttgctcaggcggcccttgtc		tgtccagcaccttcttcttg

tcgtcctcggccttgtagcg		cacgagatgacctcttgaca
agcgcgttcttggcggccac		cttctcggccaaggtgttgg
ctgagcttgcccttgagacc		tcctcttgtgctcaaactcg
cagcaccttcttcttgtcag		gggttacacacctgctccag
gagatgacctcctggcactt		ctggtacagtccgctgatga
cagcgtgttggagtccagcc		tgtccgtgaaggccacgtag
cctggtacagcccactgatg		gtagaatgccttggtctccc
ctgagccagaggctcccttg		tgtgttttctcttgaactcc
cagtgcttcatgtcggactg		caaacagggagtcgatctcc
cggcgctcttcatgttgaag		gacgtgtagaagtcgatgcc

Supplementary Table 2| Custom designs of *Hsp70* probes for smFISH.

Supplementary References

1. Torii, M., et al. Detection of vulnerable neurons damaged by environmental insults in utero. *Proc Natl Acad Sci U S A* (2017).

2. El Fatimy R*, et al.* Heat shock factor 2 is a stress-responsive mediator of neuronal migration defects in models of fetal alcohol syndrome. *EMBO Mol Med* **6**, 1043-1061 (2014).

3. Mooney SM, Siegenthaler JA, Miller MW. Ethanol induces heterotopias in organotypic cultures of rat cerebral cortex. *Cereb Cortex* **14**, 1071-1080 (2004).

4. Hashimoto-Torii K*, et al.* Roles of heat shock factor 1 in neuronal response to fetal environmental risks and its relevance to brain disorders. *Neuron* **82**, 560-572 (2014).
